# Portable Cell Tracking Velocimetry for Quantification of Intracellular Fe Concentration of Blood Cells

**DOI:** 10.3390/mi16020126

**Published:** 2025-01-23

**Authors:** Linh Nguyen T. Tran, Karla Mercedes Paz Gonzalez, Hyeon Choe, Xian Wu, Jacob Strayer, Poornima Ramesh Iyer, Maciej Zborowski, Jeffrey Chalmers, Jenifer Gomez-Pastora

**Affiliations:** 1 Department of Chemical Engineering, Texas Tech University, Lubbock, TX 79409, USA; nguyen-thuy-linh.tran@ttu.edu (L.N.T.T.); kpazgonz@ttu.edu (K.M.P.G.); 2 William G. Lowrie Department of Chemical and Biomolecular Engineering, The Ohio State University, Columbus, OH 43210, USA; choe.123@buckeyemail.osu.edu (H.C.); wu.4073@buckeyemail.osu.edu (X.W.); strayer.117@buckeyemail.osu.edu (J.S.); iyer.267@buckeyemail.osu.edu (P.R.I.); chalmers.1@osu.edu (J.C.); 3 Department of Biomedical Engineering, Cleveland Clinic, Cleveland, OH 44195, USA; zborowm@ccf.org

**Keywords:** cell tracking velocimetry, magnetic susceptibility, single-cell analysis, red blood cells, hematological characterization

## Abstract

Hematological analysis is crucial for diagnosing and monitoring blood-related disorders. Nevertheless, conventional hematology analyzers remain confined to laboratory settings due to their high cost, substantial space requirements, and maintenance needs. Herein, we present a portable cell tracking velocimetry (CTV) device for the precise measurement of the magnetic susceptibility of biological entities at the single-cell level, focusing on red blood cells (RBCs) in this work. The system integrates a microfluidic channel positioned between permanent magnets that generate a well-defined magnetic field gradient (191.82 TA/mm^2^). When the cells are injected into the chamber, their particular response to the magnetic field is recorded and used to estimate their properties and quantify their intracellular hemoglobin (Hb) concentration. We successfully track over 400 RBCs per condition using imaging and trajectory analysis, enabling detailed characterizations of their physical and magnetic properties. A comparison of the mean corpuscular hemoglobin measurements revealed a strong correlation between our CTV system and standard ultraviolet–visible (UV-Vis) spectrophotometry (23.1 ± 5.8 pg vs. 22.4 ± 3.9 pg, *p* > 0.05), validating the accuracy of our measurements. The system’s single-cell resolution reveals population distributions unobtainable through conventional bulk analysis methods. Thus, this portable CTV technology provides a rapid, label-free approach for magnetic cell characterization, offering new possibilities for point-of-care hematological analysis and field-based research applications.

## 1. Introduction

The characterization of cellular magnetic properties could play a fundamental role in numerous biomedical applications, from diagnostic procedures to therapeutic interventions [1,2]. For example, in the presence of a magnetic field gradient, the quantification of the magnetic moment of cells can be used to determine their intracellular composition, including the levels of critical metals like iron (Fe), which regulate numerous metabolic pathways essential to remaining healthy. However, traditional approaches for measuring the magnetic susceptibility of biological materials have relied on complex laboratory equipment such as Superconducting Quantum Interference Devices (SQUIDs) and specialized magnetic separation systems [3,4,5,6,7,8,9,10]. Even though these conventional magnetic susceptibility measurement systems deliver high-precision measurements, they are restricted to controlled laboratory environments, requiring extensive infrastructure and specialized operational conditions [11]. In clinical settings, automated hematology analyzers based on the Coulter principle and flow cytometry are standard for blood cell analysis and Fe and hemoglobin (Hb) quantification (in humans, approximately 75% of Fe is found in Hb) [12]. While these analyzers provide comprehensive blood cell counts and indices, they are expensive, require regular maintenance, and are confined to clinical laboratories [13,14,15,16]. Such limitations have hindered access to both routine hematological testing and advanced magnetic susceptibility measurements in resource-limited settings and field applications, where rapid and portable analysis could offer valuable diagnostic insights [13]. Specifically, portable devices can democratize access to advanced healthcare in resource-limited countries, for example, in the diagnosis and monitoring of sickle cell disease, which is much needed in sub-Saharan Africa [17]. Additionally, rapid analysis is required when testing biological samples, especially blood cells, in order to avoid contamination and sample degradation [18].

Recent advances in magnetic cell separation technologies have highlighted the importance of precise magnetic susceptibility measurements across different material types [11,19,20,21]. For instance, our cell tracking velocimetry (CTV) technology enables the real-time tracking of individual cells and particles, providing direct measurements of cellular magnetic properties without the need for cryogenic conditions [3,22,23,24]. This ability of CTV makes it particularly valuable for analyzing heterogeneous cell populations and detecting subtle variations in magnetic susceptibility at the single-cell level, providing distinct advantages over traditional bulk measurement techniques [25,26].

The fundamental principles of magnetic cell separation rely on the interaction between the target material (immunomagnetically labeled cells or unlabeled cells) and magnetic field gradients, where differences in magnetic susceptibility are the driving force for the effective separation of cellular populations [27,28]. These differences become particularly significant in applications such as blood cell analysis, where even slight variations in Hb content can produce measurable changes in magnetic behavior [14,25,26]. A recent work by Barua et al. emphasized the critical role of Fe content in the paramagnetic behavior of blood cells, which affects the magnetic separation of cells for the diagnosis and treatment of blood diseases, highlighting the need for precise susceptibility measurements in both research and clinical applications [29]. Although conventional CTV systems are more accessible than traditional magnetic measurement techniques, they still face limitations in their deployment flexibility. These systems typically require stable laboratory environments and specialized technical expertise for their operation [29]. The need for static facilities has hindered the widespread adoption of CTV in point-of-care diagnostics and field-based research, where magnetic susceptibility measurements could provide immediate and valuable insights into cellular properties [30].

Building upon these developments and addressing the current limitations, we present a portable CTV system that introduces various key advancements. For example, our novel system integrates a portable, handheld camera to perform cell tracing, replacing the traditional, complex, and expensive setup while maintaining laboratory-grade precision in a compact format. Indeed, our previous technology required significant laboratory space, as well as sensitive and expensive pieces of equipment to operate [1], whereas our new setup significantly decreased its cost by more than 10 times while allowing for portability, primarily due to advances in imaging that now allow for the tracing of micron-sized particles in microchannels with ultra-small microscopes. We demonstrate that this device is still capable of distinguishing between diamagnetic and paramagnetic materials, as well as between normal RBCs and Fe-deficient cells, by analyzing samples obtained from a variety of donors. More importantly, we continue using a simplified user interface that enables operation by personnel with minimal technical training [11,31]. Finally, our statistical analysis demonstrates that the accuracy of our new setup in determining the Hb in single cells is comparable to traditional analyses that provide an average Hb level for all the cells in the sample, proving the analytical and diagnostic power of this instrument. Thus, unlike existing portable solutions that sacrifice precision for mobility, our system maintains a measurement accuracy comparable to laboratory instruments while having a significantly reduced size and complexity.

We consider that our portable technology will have a tremendous impact in the field of hematology, iron disorders, and other related conditions that affect the magnetic susceptibility of biological materials, because it will allow for a simple, fast, inexpensive, and label-free diagnosis. Recent validation studies have demonstrated the potential impact of portable CTV technology. For instance, Weigand et al. showed the effectiveness of CTV in differentiating between normal and pathological red blood cells (RBCs) based on their magnetic signatures, while other researchers have validated its precision in quantifying cellular magnetic susceptibility under various conditions [32]. The advances in portable magnetic measurement capabilities will open up new possibilities for both research and clinical applications, particularly in resource-limited settings where traditional analytical tools are inaccessible.

This work presents a comprehensive analysis of our portable CTV device’s capabilities in quantifying magnetic susceptibility across different material categories. We address the technical challenges of maintaining measurement accuracy while enhancing portability, with a particular emphasis on optimizing magnetic field gradients. Our work contributes to the growing field of portable diagnostic and analytical tools, demonstrating the potential for precise magnetic characterization in field-based research, point-of-care diagnostics, and resource-limited settings. As the demand for portable, precise magnetic characterization tools continues to grow across various biomedical applications [33], our system represents a significant step forward in making advanced cellular analysis more accessible and practical for diverse research and clinical environments.

## 2. Materials and Methods

### 2.1. System Design and Components

**Figure 1:**
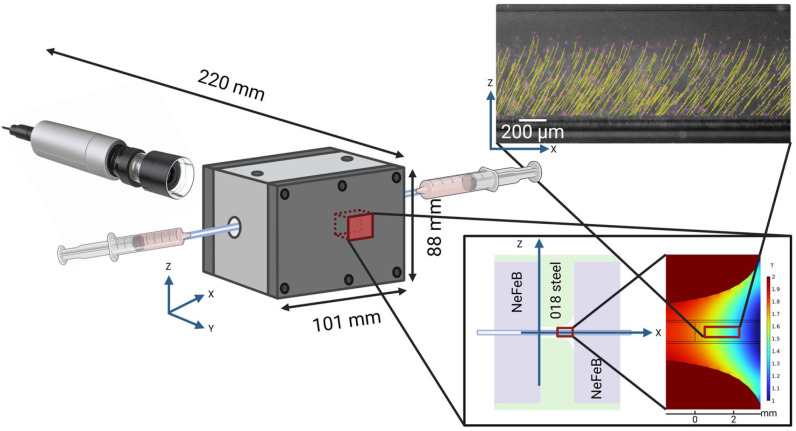
Schematic diagram of the CTV system showing the optical setup, measurement chamber dimensions, and sample trajectory analysis. Magnets are located inside the measurement chamber and an onset is provided along with the magnetic field intensity in the measurement area.

The portable CTV device comprised a magnetic chamber based on N52 neodymium magnets (K&J Magnetics) with machined 1018-steel pole pieces. The fluidic chamber consisted of square borosilicate glass tubing (1 mm inner diameter, VitroTubes, VitroCom, Mountain Lakes, NJ, USA) positioned between the pole pieces. This configuration provided a defined measurement channel while maintaining optical clarity for imaging. The sample chamber was designed to minimize wall effects on the cell/particle motion while facilitating a smooth sample flow. The imaging system utilized a portable digital microscope camera (AM73515MZTL, Dino-Lite, Torrance, CA, USA) with integrated LED illumination. The camera was mounted perpendicular to the measurement channel using a support structure that ensured stable positioning and vibration isolation (Figure 1). Image acquisition was performed at 60 frames per minute with a resolution of 2560 × 1920 pixels, corresponding to a pixel size of 1.0799 μm × 1.066 μm. Real-time particle tracking and analysis were conducted using ImageJ’s FIJI plugin with TrackMate for automated particle detection and trajectory analysis [34].

### 2.2. Magnetic Field Characterization Using Diamagnetic Beads

When particles or cells are injected into a CTV system, they experience magnetic forces in the horizontal direction and gravitational forces in the vertical direction that dictate the overall cell trajectory. The magnetic force field distribution in our new system was quantified by analyzing the particle trajectories across different positions in the channel, otherwise known as the region of interest (ROI). The magnetically and gravitationally induced velocities ($u_{m}$ and $u_{s}$) of the particles can be calculated as follows:

(1) $$u_{m}=\frac{\left(\chi_{cell}-\chi_{fluid}\right)V_{cell}}{3\pi D_{cell}\eta }S_{m}$$

(2) $$u_{s}=\frac{\left(\rho_{cell}-\rho_{fluid}\right)V_{cell}}{3\pi D_{cell}\eta }g$$

where the cell and the suspending fluid are represented by the subscripts “cell” and “fluid”, respectively, ρ is the density, the physical dimensions of the cell/particle are its diameter (D) and volume (V), η is the fluid viscosity, and the gravitational acceleration (g) is constant at 9.8 m/s^2^.

The magnetic field gradient was estimated for the ROI using characterized diamagnetic particles in a paramagnetic medium at various positions to determine the optimal conditions for particle tracking. Polystyrene microspheres (5 μm in diameter) purchased from Sigma-Aldrich and suspended in aqueous manganese (II) chloride (MnCl_2_) solutions were used in these studies. MnCl_2_ solutions were prepared at concentrations ranging from 0.025 M to 0.075 M to serve as the paramagnetic medium. The volumetric magnetic susceptibility of the MnCl_2_ solution $\chi_{MnCl_{2},aq}^{V}$ was calculated from the molar concentration of the MnCl_2_ solution, according to the following:

(3) $$\chi_{MnCl_{2},aq}^{V}=\chi_{MnCl_{2}}^{M}\left[MnCl_{2}\right]+\chi_{H_{2}O}^{V}$$

where the values (SI units) of $\chi_{MnCl_{2}}^{M}$ and $\chi_{H_{2}O}^{V}$ are 1.80 × 10^−4^ and −9.05 × 10^−6^ dm^3^·mol^−1^, respectively [35]. Furthermore, the difference in the magnetic susceptibility $\left(∆\chi \right)$ between the polystyrene particles ${(\chi }_{p})$ in SI units (−7.5 × 10^−6^) and the surrounding medium is as follows:

(4) $$∆\chi =\chi_{p}-\chi_{MnCl_{2},aq}^{V}$$

The particle concentration was maintained at approximately 10^6^ particles/mL for these tests to ensure sufficient tracking events while avoiding particle interactions. For each concentration, particle motion was analyzed within a defined ROI measuring 1.5 mm × 1.5 mm at the center of the flow channel. Video recordings were captured at 60 fpm for 120 s per measurement, with a minimum of 1000 tracked particles per condition to ensure statistical reliability. Overall, these experiments allowed us to determine the field gradient in the system, or the *S_m_* value, after introducing the magnetic velocity measured experimentally in Equation (1).

The *S_m_* value was also quantified using numerical modeling. After creating the 3D geometry of the CTV system in AutoCAD (AutoCAD^®^, version 2023) with precise dimensions, the geometry was imported into COMSOL (COMSOL Multiphysics^®^, version 6.2). The design and components of the instrument were similar to our previous device [36,37], but the magnetic components were replaced with N52 neodymium magnets to enhance the magnetic field strength, and the measurement view was shifted to the opposite side. The magnetic field was analyzed using the “Magnetic Fields, No Current (mfnc)” module. All material properties, including those for air, aluminum, Low-Carbon Steel 1018, and N52 magnets, were sourced from the COMSOL material library. To match each material’s magnetic properties using the options available in the mfnc module, the N52 magnets were defined with the remanent flux density option, maintaining the magnetization direction consistent with the previous device design. Low-Carbon Steel 1018 was modeled using the **B**–**H** Curve option, while other materials were specified using the relative permeability option.

### 2.3. Red Blood Cell Analysis

The CTV system was also employed to measure the hematological indices in human RBCs. Human whole-blood samples (35 ± 5 mL) were collected from four volunteers using ethylenediaminetetraacetic acid (EDTA) tubes upon informed consent according to the Institutional Review Board (IRB)-approved protocol of the Texas Tech University Health Science Center (Protocol number IRB: L22–L274). These samples were separated into two aliquots, with each designated for whole-blood analysis and RBC analysis. The RBC aliquots were further processed and washed in phosphate-buffered saline (PBS) to separate the RBCs from the plasma (three times, via 2000× *g* centrifugation for 5 min). The washed RBCs were divided into two fractions, one for CTV analysis and the other for reference measurements. For CTV measurements, the RBCs were prepared for analysis by oxidation with a sodium nitrite (NaNO_2_) solution to generate methemoglobin-containing RBCs (metHb-RBCs), as previously published [38,39]. The cell suspension was diluted to 1 × 10^6^ cells/mL and introduced into the CTV instrument. Sample channels were sealed using Hamilton 1–1 valves (Hamilton Company, Reno, NV, USA) to minimize flow disturbances. After a 60 s equilibration period, cell movement was recorded at room temperature (22 ± 2 °C). Image sequences were analyzed using custom software to determine the magnetic and settling velocities, which were then converted into susceptibility measurements and, subsequently, into Hb concentrations [40].

Additionally, Hb quantification was performed in the samples using a ultraviolet–visible (UV–Vis) spectrophotometer, as this is the approach usually performed in the clinic to determine the Hb levels in blood and for anemia diagnosis [41]. RBC samples for spectrophotometry were prepared by performing cell lysis in deionized water at a four-fold dilution. After vortexing, the samples were incubated at 4 °C for 30 min to ensure complete lysis, followed by centrifugation (2000× *g*, 5 min). The Hb concentration in the supernatant was measured using the Agilent Cary 60 UV–Vis spectrophotometer (Agilent Technologies Inc., Santa Clara, CA, USA). The samples were diluted to achieve absorbance values between 0.1 and 1.0 at the Q bands (~523 nm) for optimal accuracy. Each sample underwent triplicate testing to calculate the concentrations of Hb using the equations developed by Winterbourne [42].

We needed a quantification of the cell concentration in the sample to compare our CTV analysis (Hb quantification per cell) to the Hb quantification via spectrophotometry. The RBC count and cell size distribution were measured using a B23005 Multisizer 4e Coulter Counter (Beckman Coulter, Brea, CA, USA). Multiple CTV runs were performed to collect data for statistical significance. Correlation studies between CTV measurements and reference methods included comparing directly measured and calculated intracellular Hb. The analysis accounted for concentration differences between the CTV measurements and spectrophotometric analysis.

## 3. Results

### 3.1. S_m_ Quantification Using Diamagnetic Particles

**Figure 2:**
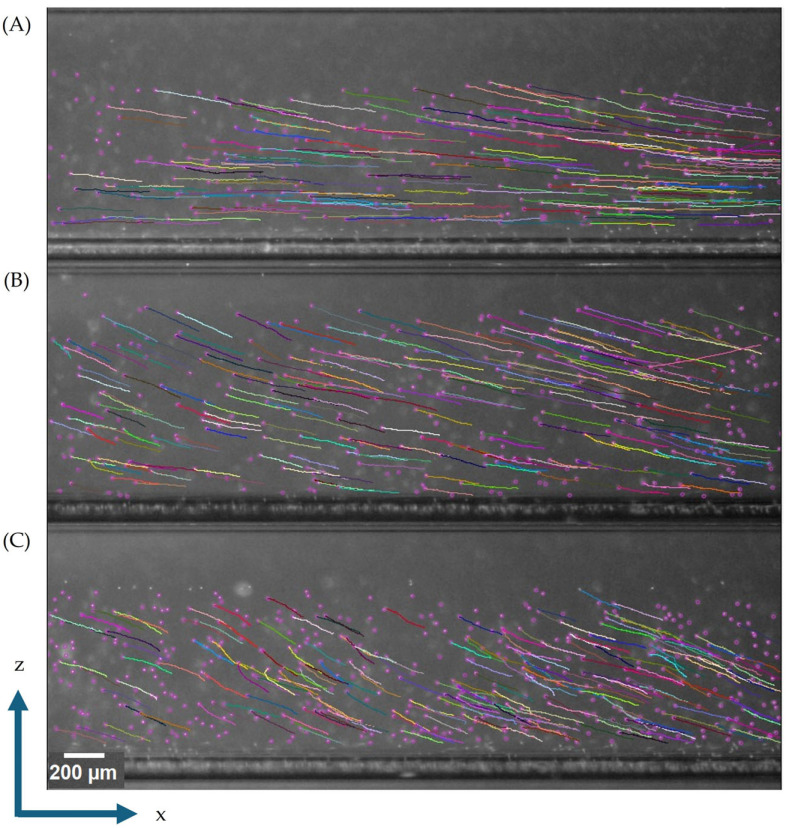
Representative particle trajectories of polystyrene beads in MnCl_2_ solutions at different concentrations: (**A**) 0.025 M, (**B**) 0.05 M, and (**C**) 0.075 M.

The magnetic energy gradient (*S_m_*) was experimentally determined by tracking polystyrene microspheres suspended in paramagnetic solutions of various concentrations inside the CTV device. Initial imaging was performed across multiple sections of the CTV device, and the section with the most uniform particle trajectories was selected for subsequent analyses, as it corresponded to the highest and most uniform magnetic energy gradient. Figure 2 illustrates the particle trajectories in this section for various paramagnetic solution concentrations. As the concentration increases, the magnetic susceptibility difference between the fluid and the particles grows, leading to an increase in the particle magnetic velocity in the horizontal direction, which is evident from the steeper trajectory angles. The multi-colored tracks represent individual cell/particle paths, illustrating the system’s ability to simultaneously monitor and distinguish hundreds of entities while maintaining a single-cell and/or single-particle resolution. It should be noted that the different trajectories or paths observed among the particles for each condition is due to the variability of the magnetic field energy gradient *S_m_* at varying locations in the channel. As stated above, the current design was optimized around the principle that, within the microscopic viewing region, the product of field **B** and grad **B** generates a nearly parallel magnetic force line of a constant magnitude, however, the gradient is not exactly the same across the entire viewing region, which affects the particle paths observed at different locations. Furthermore, the particles/cells are not all homogeneous and have a distribution of properties (size, density, and magnetic susceptibility) that can also greatly affect their motion in the field of view and can lead to different trajectories among them.

**Figure 3:**
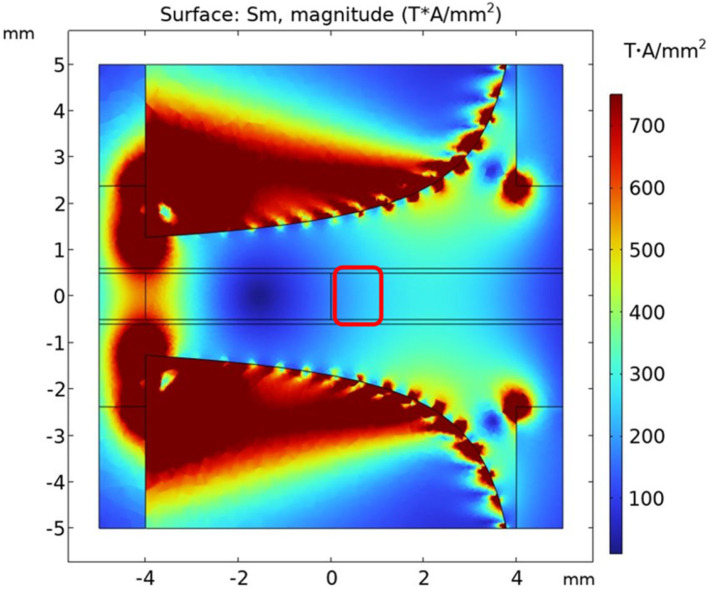
COMSOL-simulated magnetic energy gradient. The red rectangle in the channel presents the ROI used for tracing the particles in our experiments.

Using velocity data extracted from the particle tracks, *S_m_* was calculated for each trajectory using Equation (1). The experimental *S_m_* for the selected section was then determined as the average *S_m_* across all tracks obtained at different fluid concentrations, yielding a value of 191.82 TA/mm^2^. This experimental result aligns well with the simulated magnetic energy gradient obtained from COMSOL simulations, as presented in Figure 3. Note that, in Figure 3, our experimentally selected ROI is presented, with dimensions of approximately 1.5 × 1.5 mm^2^ in width and height.

### 3.2. RBC Analysis and Hb Quantification

**Figure 4:**
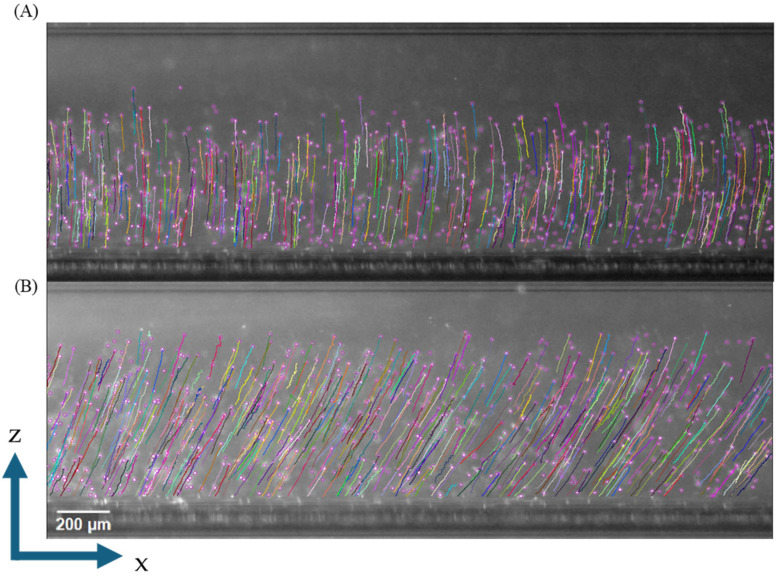
Representative cell trajectories of (**A**) oxyhemoglobin-containing RBCs and (**B**) methemoglobin-containing RBCs inside the CTV device. Note the different trajectories of both cell types, with the non-magnetic (diamagnetic) oxyhemoglobin cells moving only downwards due to gravity and with the paramagnetic metHb-RBCs moving in both directions (horizontally and vertically) due to the effect of the magnetic and gravitational fields.

The microscale analysis capabilities of our CTV system enable the direct visualization and tracking of individual RBC trajectories under applied magnetic fields. Figure 4 shows representative cell tracking data from a single donor sample, comparing oxyhemoglobin-containing (Figure 4A) and metHb-RBCs (Figure 4B). The system successfully tracked 421 individual oxyhemoglobin RBC trajectories and 472 metHb-RBC trajectories, demonstrating robust cell detection and tracking capabilities. The distinct trajectory patterns reflect the different magnetic properties of these Hb states, with the metHb-RBCs exhibiting steeper trajectories due to their paramagnetic response, consistent with previous magnetic characterization studies [1,25,39,41]. On the contrary, oxyhemoglobin RBCs containing Hb molecules that are essentially diamagnetic in water only settled down in the CTV due to the gravitational force, as the cells experienced negligible magnetic forces in this state. As happened before with the particles, not all the cells displayed the same trajectory within the channel for each condition, which was expected due to their different properties and the non-constant *S_m_* value across the entire viewing region.

**Figure 5:**
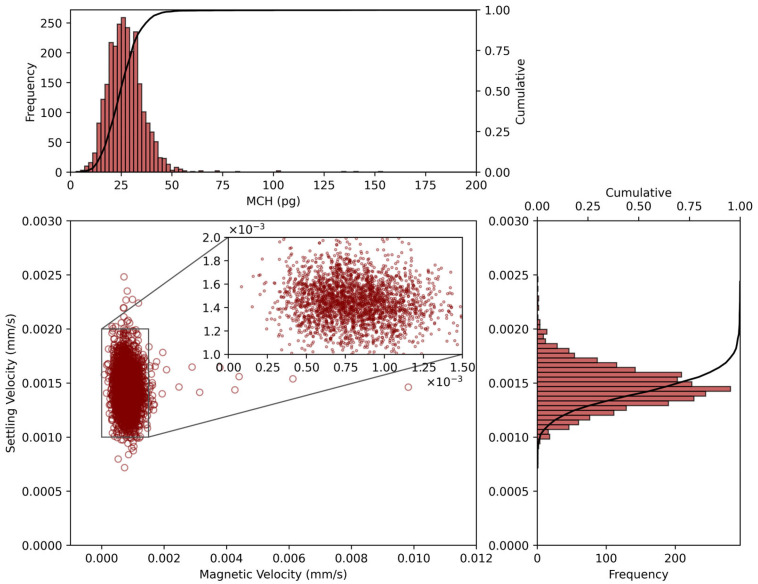
Distribution plots showing the relationship between MCH content (pg of Hb per cell), magnetically induced velocity (mm/s), and settling velocity (mm/s) measured from a representative RBC sample. Top panel: MCH distribution with cumulative frequency. Bottom panels: scatter plot of settling velocity versus magnetically induced velocity (**left**) and corresponding settling velocity distribution (**right**).

Through the precise tracking of these cell trajectories in the measurement chamber, detailed characterizations of individual RBCs in terms of their magnetic properties were performed. The high number of tracked cells (>400 per condition) ensured statistical reliability in the subsequent analysis. Single-cell analysis revealed distinctive patterns in magnetic behavior, quantified through the distribution of the cell magnetic susceptibility, which was subsequently employed to calculate the mass of Hb per cell, called the mean corpuscular hemoglobin (MCH), which is in the range of 26–33 pg under normal conditions. As shown in Figure 5 for a representative sample, the MCH distribution exhibited a characteristic typical pattern centered at around 30 pg, with cell populations predominantly falling within the 20–50 pg range. This distribution profile aligns well with the expected physiological values for healthy donors, with the cumulative frequency analysis showing approximately 90% of cells below 45 pg, consistent with established clinical reference ranges [43].

Building upon the trajectory analysis, a detailed examination of cell movement parameters provided insights into the relationship between magnetic properties and cellular characteristics. A scatter plot comparing magnetically induced velocity and settling velocity (Figure 5, bottom panels) reveals a defined correlation pattern, with magnetic velocities primarily distributed in the range of 0–0.002 mm/s and settling velocities spanning 0.0005–0.0025 mm/s. The majority of cells cluster within a characteristic region (0.001–0.002 mm/s for both magnetic and settling velocity), indicating a consistent measurement precision across the RBC population. This clustering pattern suggests uniform magnetic properties as well as size and density among the RBCs, with the observed variations reflecting the natural biological diversity in cellular Hb content, cell size, and other intracellular components, such as water. The settling velocity distribution (right panel) shows a normal pattern, in agreement with our previous observations with the bulky CTV device, providing an additional validation of the measurement consistency [41].

**Figure 6:**
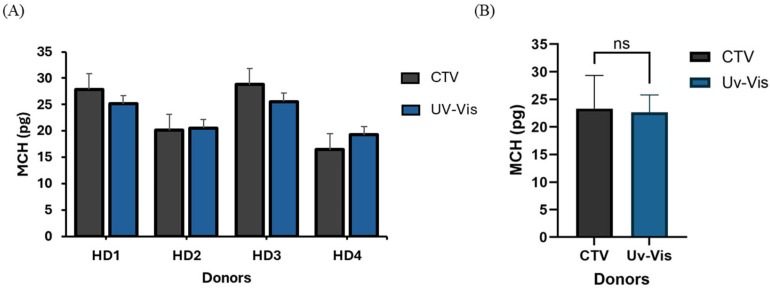
MCH content analysis by CTV and UV–Vis spectrophotometry. (**A**) Individual MCH values across donors HD1–HD4. (**B**) Statistical comparison of mean MCH values (pg) between methods. Error bars represent standard deviation; ns: not significant (paired *t*-test, *p* > 0.05).

To establish Hb measurement accuracy, comparative analyses between the portable CTV system and standard UV–Vis spectrophotometry on multiple human donor blood samples (HD1–HD4) were conducted. Individual donor comparisons, presented in Figure 6A, demonstrated strong agreement between the two methods. The analysis of donor HD3 revealed the highest MCH values (CTV: 28.81 pg; UV–Vis: 25.53 pg), while HD4 showed comparatively lower values (CTV: 20.11 pg; UV–Vis: 19.28 pg). Potential differences in the values obtained with each method could have been caused by the different approaches employed in these analyses, as we indirectly quantified Hb by measuring other physical properties (in the CTV device, we quantified Fe content via magnetic susceptibility, which was later related to Hb distribution, and in the UV-Vis spectrophotometry, we quantified changes in the color of the solution after the Hb was released from the cells), as well as the fact that CTV measures the Hb distribution in single cells (we quantified Hb in ~1000 cells), whereas UV–Vis spectrophotometry measures the Hb in the solution after performing cell lysis. This means that UV–Vis spectrophotometry can potentially detect free Hb in a sample not contained within RBCs, which is not detectable with CTV. Nevertheless, both approaches provided very similar Hb levels for the samples. The consistency in these measurement trends across donors with varying MCH levels demonstrates the system’s reliability across different physiological conditions and its potential to accurately detect anemias. A statistical evaluation of aggregate data (Figure 6B) confirmed measurement equivalence between the portable CTV device and laboratory-based UV–Vis spectrophotometry. The mean MCH values obtained via CTV (23.1 ± 5.8 pg) showed no statistically significant difference from the UV–Vis measurements (22.4 ± 3.9 pg) (paired *t*-test, *p* > 0.05). The comparable standard deviations between methods indicate a similar measurement precision, as evidenced by the overlapping error bars in Figure 6B, and the diagnostic power of our instrument in detecting low Hb values in blood, and, hence, its ability to diagnose anemias. This statistical equivalence is particularly noteworthy given that the CTV system provides a single-cell resolution compared to the bulk measurements of UV–Vis spectrophotometry.

The integration of magnetophoresis, particle tracing, and the estimation of magnetic velocity and settling velocity under controlled magnetic fields allows for the determination of MCH and other potential RBC indices, thus opening the door for a comprehensive framework for single-cell characterization. The observed correlations between magnetic susceptibility and the Hb level and Hb state in cells enable the precise quantification of cellular properties at the microscale level, achieving a measurement precision comparable to conventional laboratory techniques while providing additional insights through single-cell analysis. This high-resolution analysis capability provides detailed insights into RBC population heterogeneity not accessible through conventional bulk measurements, offering new possibilities for hematological research and diagnostic applications. It should be noted that the magnetic susceptibilities of individual RBCs are the data that we use to obtain Hb concentration distributions. If there are biological markers that affect the Hb levels or the Hb oxidation state, as well as the cell size and density (such as metabolic activity, membrane integrity, etc.), we could indirectly assess these using the obtained magnetic susceptibility and settling behavior, increasing the analytical capabilities of our system.

## 4. Conclusions

This work demonstrates the successful development and validation of a portable CTV system that advances the accessibility of cellular magnetic property measurements. The transition from traditional laboratory-bound instruments to a portable format represents a significant step forward in magnetic cell analysis, with a high measurement precision. The ability to simultaneously track and image hundreds of individual cells under specific magnetic field conditions at a high resolution clearly demonstrates that portability does not compromise analytical capabilities. This is further validated by the strong statistical agreement between our portable CTV measurements and standard laboratory UV–Vis spectrophotometry for Hb measurements in single cells. Key technical achievements include the characterization and optimization of the magnetic field gradient (*S_m_* = 191.82 TA/mm^2^), allowing for single-cell analysis and the development of robust cell tracking protocols that maintain precision outside controlled laboratory environments. The precise calibration of the magnetic field gradient using polystyrene microspheres in paramagnetic solutions of varying concentrations ensured reliable and reproducible measurements of human cells across different experimental conditions. These advancements effectively bridge the gap between traditional, resource-intensive magnetic susceptibility measurement techniques and the growing need for portable, accessible solutions. The system can provide a single-cell resolution with a measurement accuracy comparable to laboratory-grade instruments, showing that portable devices can achieve sophisticated analytical capabilities without sacrificing precision. Even though we performed our experiments in a controlled environment in our laboratory, we are confident about the stability and convenience of the system in actual field application scenarios, as this device only requires the handheld camera, the channel, the magnets, and two syringes for operation, which are very robust. The system’s benefits, especially in low-resource settings or at the point-of-care, rely on its portability, low cost, recyclability (the magnets can operate for decades without any maintenance), and speed (samples can be measured in 5 min). Thus, the successful implementation of this portable CTV system establishes a new paradigm for field-deployable magnetic cell analysis. Its ability to detect variations in RBC magnetic properties without requiring complex laboratory infrastructure opens up new possibilities for point-of-care diagnostics and field research applications. The demonstrated capacity to distinguish between oxyhemoglobin and metHb-RBCs through their distinct trajectory patterns showcases the system’s potential for detailed cellular characterization in various physiological states. Its proven reliability across diverse physiological conditions, combined with its portable nature, makes it particularly valuable in resource-limited settings where traditional analytical tools may be not accessible.

Looking forward, this technology could be adapted for analyzing other cell types and biological systems where magnetic properties and Fe levels serve as important biomarkers. For example, we could study the magnetic properties and Fe levels of white blood cells (macrophages and monocytes, involved in Fe recycling [39,44,45,46,47], and lymphocytes, which can change their Fe content depending on the extracellular Fe concentration and whose Fe levels are strongly correlated with changes in their cellular activation characteristics [48]), micro- and nanovesicles, which can be involved in Fe trafficking across membranes [49], as well as tumor cells, in order to decipher the role of Fe levels in cancer initiation, progression, and metastasis, as well as to study the success of ferroptosis-based therapies against their growth and dissemination [50,51,52,53,54,55,56,57,58,59,60,61,62]. Thus, our system’s capability to conduct real-time analysis of cellular magnetic properties and intracellular Fe concentrations could prove valuable for monitoring disease progression, therapeutic response, and cellular alterations in various pathological conditions. This advancement in portable microscale analytical technology contributes to democratizing sophisticated cellular analysis, with potential applications ranging from field-based diagnostics to remote healthcare settings, ultimately expanding the reach of advanced cellular characterization techniques beyond traditional laboratory boundaries. Future developments could focus on enhancing the system’s capabilities to automate the analysis of other blood cell populations and adapting it for specific clinical applications.

## Data Availability

The original contributions presented in this study are included in the article. Further inquiries can be directed to the corresponding author.
